# Determinants of place of death among individuals with cardiovascular disease: A Saudi sub-national register study

**DOI:** 10.1371/journal.pone.0335669

**Published:** 2025-10-29

**Authors:** Ahmed Alshehri, Faleh Alyazidi, Mária Bartušová, Mayssa Rekhis, Leo Stockfelt, Laith Hussain-Alkhateeb

**Affiliations:** 1 Department of Public Health, Faculty of Health Sciences, Al-Qunfudah, Umm Al-Qura University, Makkah, Saudi Arabia; 2 School of Public Health and Community Medicine, Institute of Medicine, Sahlgrenska Academy, University of Gothenburg, Gothenburg, Sweden; 3 Department of Public Health, College of Health Sciences at Al-Leith, Umm Al-Qura University, Al-Leith, Kingdom of Saudi Arabia; 4 Institute of Epidemiology and Prevention, Faculty of Public Health, Slovak Medical University in Bratislava, Bratislava, Slovakia; 5 Department of Occupational and Environmental Medicine, Sahlgrenska University Hospital, Gothenburg, Sweden; 6 Population Health Research Section, King Abdullah International Medical Research Centre, Riyadh, Saudi Arabia; Jazan University College of Applied Medical Science, SAUDI ARABIA

## Abstract

Understanding the place of cardiovascular disease (CVD) deaths is essential for identifying vulnerable populations and guiding health system interventions. This study investigated the demographic and clinical determinants of the place of death among individuals with CVD in Makkah City, Saudi Arabia. We analyzed 9,652 CVD death records from two major hospitals (2018−2023) for decedents aged ≥18 years, including the place and year of death, sex, age, citizenship, and causes of death (ICD-10). Chi-square tests assessed associations and multivariable logistic regression identified independent predictors of out-of-hospital deaths. Out-of-hospital deaths accounted for 90.0% of all CVD deaths. Within each age group, the proportion of deaths that occurred out-of-hospital was highest among the youngest (18–45 years, 92.8%) and oldest (76 + years, 90.1%) age groups, with lower levels among middle-aged adults (46–60 years, 88.2%; 61–75 years, 86.6%). By citizenship, 92.6% of non-Saudis (n = 4,273), 90.8% of pilgrims (n = 2,248), and 84.0% of Saudis (n = 2,150) died outside hospital settings. There were significant associations between place of death and age, citizenship, ethnicity, and cause of death (all p < 0.001). Out-of-hospital deaths declined from 93.6% in 2018 to 77.2% in 2023, but sudden cardiac death (SCD) had the highest proportion (93.6%). Adjusted analyses showed higher odds of out-of-hospital death for non-Saudis (aOR = 2.05, 95% CI: 1.47–2.85) and pilgrims (aOR = 1.91, 95% CI: 1.38–2.66) compared with Saudis. SCD was strongly associated with out-of-hospital death compared with ischemic heart disease (aOR = 46.74, 95% CI: 37.35–58.48). Despite recent declines, most CVD deaths occurred outside hospitals, particularly among non-Saudis and in the youngest and oldest age groups. The predominance of SCD especially in out-of-hospital cases, may reflect diagnostic limitations and possible misclassification when clinical assessment is unavailable. Strengthening medical certification processes, improving access to timely emergency care, and exploring the role of factors such as health insurance, indirect costs, and health literacy in future research are priorities for reducing preventable CVD mortality.

## Introduction

Cardiovascular diseases (CVD) are the leading cause of death (CoD) globally, being responsible for approximately 18 million deaths annually, which accounts for 32% of all global fatalities [1]. The majority of these deaths (85%) result from heart attacks and strokes [2]. In Saudi Arabia, CVD affects approximately 1.6% of individuals aged 15 and older, with prevalence increasing steadily with age and reaching 11% among those aged 65 and older [3]. When disaggregated by sex, males exhibit a higher prevalence 1.9% compared to females 1.4%. Regionally, the Makkah region has the highest reported CVD prevalence at 1.9% followed by Riyadh at 1.7% [3]. The specific focus on CVD deaths in this study arises from the unique challenges associated with the high proportion of these deaths occurring in out-of-hospital settings [4,5]. Due to limited clinical documentation, out-of-hospital deaths may be more prone to misclassification and underreporting, potentially affecting the reliability of mortality statistics used in public health decision-making [6–8].

The place of death is an important public health indicator that reflects healthcare system capacity, accessibility, and end-of-life care practices [9]. While some individuals and families may prefer home-based care for reasons of comfort, familiarity, or cultural beliefs, hospital deaths can provide critical access to emergency interventions, particularly for conditions like CVD, where timely treatment is often essential for survival. International studies have shown substantial variation in place of death patterns by cause, with distinct profiles reported for CVD, cancer, dementia, and pediatric diseases [10–13]. These patterns are often shaped by sociodemographic and clinical factors such as age, sex, and CoD [9,11,14–19]. Despite this, there is limited research examining the place of death patterns, specifically in Saudi Arabia.

In countries like Saudi Arabia, which has a highly heterogeneous population characterized by different cultures, beliefs, socioeconomic statuses, and disease patterns [20], the determinants of place of death might be complex. This complexity is particularly evident in Makkah, where more than 2.5 million pilgrims from around the world gather annually for Hajj, which can last up to 30–45 days, while millions more arrive year-round for “Umrah” (a milder cluster of pilgrims). Most pilgrims are over 50 years old and can have preexisting comorbidities [21], which may contribute to a distinctive demographic and clinical profile relevant to healthcare planning and mortality patterns. In response, the Saudi Ministry of Health establishes seasonal healthcare infrastructure, including temporary hospitals and thousands of deployed healthcare workers who provide free emergency and critical care services during the Hajj period [22,23]. These unique factors highlight the importance of conducting context-specific studies to better understand place of death patterns in such a dynamic and demographically diverse setting.

Exploring the demographic and clinical determinants that influence the place of death is crucial for developing effective healthcare policies and practices in Saudi Arabia. With this in view, the present study aims to investigate the distribution and determinants of the place of death among a heterogeneous population sampled from Makkah City and determine its association with demographic and clinical factors.

## Methods

### Study population and context

This retrospective, register-based study focuses on deceased individuals with CVD, identified from the CVD mortality registry of two major hospitals (Al-Noor Specialist Hospital and King Faisal Hospital) in Makkah City, Saudi Arabia. Both hospitals are key healthcare providers offering medical services to approximately 2.5 million Saudi citizens and non-Saudi residents, as well as millions of pilgrims visiting Makkah City throughout the year [24]. Al-Noor Specialist Hospital is a 500–550 bed tertiary referral and teaching hospital that delivers specialized cardiovascular and emergency care to residents and visitors across the Makkah region [25,26]. The second is King Faisal Hospital, which is a general hospital providing routine and acute care services to a broad catchment area and both hospitals reported a total of 9,652 CVD-related deaths between 2018 and 2023. Data from these two hospitals serve as the sole source for this study due to their extensive patient coverage and comprehensive data collection.

In recent years, Saudi Arabia has transitioned from a centralized to a decentralized healthcare system, forming regional health clusters that serve defined populations of approximately one million people each. These clusters integrate primary care centers, general hospitals, and specialized services under a unified administrative system, aiming to enhance continuity of care, service efficiency, and accessibility. The Ministry of Health has shifted from a governing body to a regulatory and administrative entity, overseeing healthcare policies and quality standards rather than directly managing services [27]. Healthcare delivery is now increasingly managed by regional clusters, other governmental bodies, and private sector entities, reflecting a move toward public-private partnerships. Additionally, the recent expansion of medical insurance has begun to reshape healthcare access and utilization, influencing population health outcomes [28].

The cosmopolitan population served by healthcare institutions in Makkah encompasses Saudi citizens, non-Saudi residents (e.g., expatriate workers), and a transient population of pilgrims from around the globe, creating a unique mix of culture, ethnicity, and socioeconomic status. The non-Saudi resident population includes individuals from different ethnic groups, representing a variety of languages, health beliefs, and healthcare-seeking behaviors. Additionally, Makkah’s population expands significantly during the Hajj and Umrah seasons, with millions of national and international pilgrims arriving throughout the year, further contributing to the city’s demographic and healthcare complexities.

### Data collection and management

The retrieved death data included variables capturing demographic, clinical, and year of death (2018–2023). Demographic variables comprised sex (male, female), age (grouped into 18−45, 46−60, 61−75, and ≥76 years), citizenship status, which was classified based on hospital administrative records into mutually exclusive categories: Saudi citizen, non-Saudi resident, and pilgrim. The “pilgrim” category is typically registered as a distinguished category in Saudi Arabia (i.e., neither resident (non-Saudi) nor Saudi citizen but technically identifies those who temporarily visit Saudi Arabia for pilgrimage). Ethnicity (Arab and non-Arab) was determined based on recorded nationality. The CoD was classified according to the 10th version of the International Classification of Diseases (ICD-10) into ischemic heart disease, sudden cardiac death (SCD), hypertensive diseases, and stroke. For out-of-hospital deaths, the CoD was primarily determined based on physician-reported findings for individuals declared dead on arrival or recorded through emergency medical services documentation. In many such cases, especially when detailed medical records were unavailable, physicians relied on prior diagnoses or clinical judgment to assign causes of death. Although the WHO introduced ICD-11 in 2022, ICD-10 remained the standard classification system in hospital registers during the study period. The outcome variable was the place of death, which was classified as either in-hospital or out-of-hospital. All CVD-related deaths recorded in hospital registers were included in the study without further exclusion based on demographic or clinical characteristics. However, the data for 2023 covered only two months, which accounted for the smaller number of cases in that year. No other variables were available in the registers.

Due to low frequencies, stroke, and hypertensive diseases were combined with ischemic heart diseases in the regression analysis. In the multivariable regression analysis, ischemic heart diseases were used as the reference group for the CoD variable because ischemic heart diseases are often more adequately diagnosed and managed compared to SCD, which qualifies for a more adequate reference group [29]. Using it as the reference group facilitates a more valid comparison of the likelihood of out-of-hospital deaths among less prevalent or less clinically managed heart conditions.

### Statistical analysis

Frequencies and percentages were calculated to describe demographic factors, CoD, and place of death. Chi-square tests were performed to assess the initial associations between the place of death (in-hospital vs. out-of-hospital) and the other categorical variables. A Multivariable logistic regression analysis was conducted to examine associations between the place of death and other predictors. Variables considered as potential confounders were identified prior to the data collection through a review of relevant literature, expert opinion, and discussions among the study team. Based on the data availability, ‘age’, ‘year of death’, ‘ethnicity/ citizenship’ and the ‘CoD’ were potential confounders to assess. To construct the adjusted regression model, we applied a forward stepwise approach, which allowed us to build a parsimonious model by sequentially adding only those confounders that provided a significant improvement in model fit. This approach was chosen given the limited number of potential confounders, the emphasis on retaining only those with theoretical and empirical relevance, and the need to balance model simplicity with interpretability. The likelihood ratio test was employed to assess whether each added variable significantly improved the model fit. Statistical significance for inclusion was set at *P* < 0.1, and *P* < 0.05 was required for retention in the final adjusted model. Multicollinearity was assessed before variable inclusion, and only variables meeting theoretical and statistical relevance were retained. To assess the robustness of the findings, we conducted a sensitivity analysis excluding 2023, as this year contained only two months of data. All analyses were conducted using the software package Stata (version 18.0).

### Ethical approval

This study was conducted in accordance with the Declaration of Helsinki. Ethical approval was obtained from the Institutional Review Board at Makkah Health Affairs, Saudi Arabia (IRB Number: H-02-K-076-1122-842). The data were accessed for research purposes on 15 May 2023 and were fully anonymized before being made available to the research team. As the study involved a retrospective analysis of de-identified data, informed consent was not required, and this was approved by the ethics committees. Additional ethical approval was granted by the Swedish Ethical Review Authority (Ethix; Approval #2024-01733-01) as part of the data analysis was conducted in Sweden, where some researchers are affiliated.

## Results

**Fig 1:**
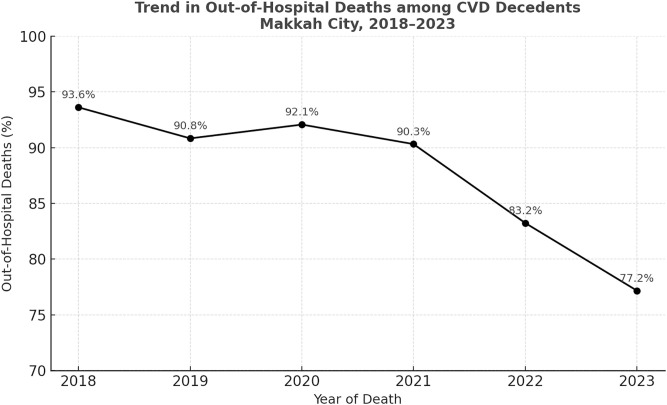
Trend in out-of-hospital deaths among cardiovascular disease (CVD) decedents, Makkah City (2018–2023).

Table 1 displays the characteristics of 9,652 CVD decedents by place of death, along with chi-square test results for categorical variables in our dataset. Among individuals aged 18–45 years, 92.8% of deaths occurred out of hospital, followed by 90.1% among those aged 76 and older., with a significant association between age group and place of death (p < 0.001). The proportion of out-of-hospital deaths was similar between males (90.1%) and females (89.5%), with no significant association between sex and place of death (p = 0.35). Out-of-hospital deaths showed a decreasing trend over time, from 93.6% in 2018 to 77.2% in 2023 (Fig 1), and this trend was statistically significant (p < 0.001). Non-Saudi residents (92.6%), pilgrims (90.8%), and non-Arab residents (92.2%) had higher proportions of out-of-hospital deaths compared to Saudi citizens (84%) and Arab residents (85.5%), with a significant association between citizenship, ethnicity, and place of death (p < 0.001). SCD predominantly occurred out-of-hospital (93.6%), whereas ischemic heart diseases (63.2%), hypertensive diseases (70.4%), and strokes (94.3%) were more often in-hospital, with a significant association between CoD and place of death (p < 0.001).

**Table 1:** Characteristics of 9,652 CVD decedents by place of death, Makkah City, Saudi Arabia, 2018 to 2023.

Characteristics	Place of death				Total		P value
	In-hospital		Out-of-hospital				
	No	%	No	%	No	%	
Age group (years)							< 0.001
18-45	100	7.2	1,299	92.8	1,399	14.5	
46-60	253	12.2	1,823	87.8	2,076	21.5	
61-75	369	10.4	3,183	89.6	3,552	36.8	
76+	259	9.9	2,366	90.1	2,625	27.2	
Sex							0.35
Female	401	10.5	3,412	89.5	3,813	39.5	
Male	580	9.9	5,259	90.1	5,839	60.5	
Year of death							< 0.001
2018	142	6.4	2,081	93.6	2,223	23.0	
2019	209	9.2	2,070	90.8	2,279	23.6	
2020	131	7.9	1,520	92.1	1,651	17.1	
2021	135	9.7	1,259	90.3	1,394	14.4	
2022	322	16.8	1,599	83.2	1,921	19.9	
2023	42	22.8	142	77.2	184	1.9	
Citizenship							< 0.001
Saudi citizen	410	16.0	2,150	84.0	2,560	26.5	
non-Saudi resident	343	7.4	4,273	92.6	4,616	47.8	
Pilgrim	228	9.2	2,248	90.8	2,476	25.7	
Ethnicity							< 0.001
Arab	490	14.5	2,889	85.5	3,379	35.0	
Non-Arab	491	7.8	5,782	92.2	6,273	65.0	
CoD							< 0.001
Ischemic heart diseases	196	63.2	114	36.8	310	3.2	
SCD	582	6.4	8,531	93.6	9,113	94.4	
Hypertensive diseases	38	70.4	16	29.6	54	0.6	
Stroke	165	94.3	10	5.7	175	1.8	
Total	981	10.0	8,671	90.0	9,652	100	

The results from the multivariable logistic regression model are presented in Table 2. Compared to younger adults (18–45 years), the odds of dying out-of-hospital were significantly lower for middle-aged individuals 46–60 years (aOR: 0.58; 95% CI: 0.43–0.78) and older adults 61–75 years (aOR: 0.65; 95% CI: 0.49–0.86). However, the oldest age group, 76 + years (aOR: 0.76; 95% CI: 0.57–1.02), showed no statistically significant difference. Males tended to be more likely to die out-of-hospital compared to females, but this result was not statistically significant (aOR: 1.14; 95% CI: 0.99–1.33). Significant yearly decreases in out-of-hospital deaths were observed in 2019 (aOR: 0.69; 95% CI: 0.55–0.86), 2021 (aOR: 0.70; 95% CI: 0.54–0.91), 2022 (aOR: 0.38; 95% CI: 0.30–0.46), and 2023 (aOR: 0.26; 95% CI: 0.18–0.39) compared to 2018, while 2020 showed no significant change (aOR: 0.84; 95% CI: 0.65–1.08). Non-Saudi residents (aOR: 2.05; 95% CI: 1.47–2.85) and pilgrims (aOR: 1.91; 95% CI: 1.38–2.66) were significantly more likely to die out-of-hospital compared to Saudi citizens. However, no significant difference in out-of-hospital deaths was observed between Arab and non-Arab residents (aOR: 1.29; 95% CI: 0.95–1.74). Furthermore, individuals with SCD were significantly more likely to die out-of-hospital (aOR: 46.74; 95% CI: 37.35–58.48) compared to those with ischemic heart diseases.

**Table 2:** Crude and adjusted associations between decedent characteristics and place of death, Makkah City, Saudi Arabia, 2018 to 2023.

Characteristics	Crude OR (95% CI)	Adjusted OR (95% CI)	P-value
Age group (years)			
18-45	*Ref*	*Ref* ^*a*^	*Ref*
46-60	0.55 (0.44–0.71)	0.58 (0.43–0.78)	< 0.001
61-75	0.66 (0.53–0.84)	0.65 (0.49–0.86)	< 0.001
76+	0.70 (0.55–0.89)	0.76 (0.57–1.02)	0.07
Sex			
Female	*Ref*	*Ref* ^*b*^	*Ref*
Male	1.07 (0.93–1.22)	1.14 (0.99–1.33)	0.07
Year of death			
2018	*Ref*	*Ref* ^*c*^	*Ref*
2019	0.68 (0.54–0.84)	0.69 (0.55–0.86)	< 0.001
2020	0.79 (0.62–1.01)	0.84 (0.65–1.08)	0.18
2021	0.64 (0.50–0.81)	0.70 (0.54–0.91)	0.01
2022	0.34 (0.28–0.42)	0.38 (0.30–0.46)	< 0.001
2023	0.23 (0.16–0.34)	0.26 (0.18–0.39)	< 0.001
Citizenship			
Saudi citizen	*Ref*	*Ref* ^*d*^	*Ref*
Non-Saudi resident	2.38 (2.04–2.77)	2.05 (1.47–2.85)	< 0.001
Pilgrim	1.88 (1.58–2.23)	1.91 (1.38–2.66)	< 0.001
Ethnicity			
Arab	*Ref*	*Ref* ^*e*^	*Ref*
Non-Arab	1.99 (1.75–2.28)	1.29 (0.95–1.74)	0.10
CoD			
Ischemic heart diseases	*Ref*	*Ref* ^*f*^	*Ref*
SCD	41.77 (33.86–51.54)	46.74 (37.35–58.48)	< 0.001

^a^adjusted for citizenship, and CoD.

^b^adjusted for age, citizenship, and CoD.

^c^adjusted for age, citizenship, and residents’ ethnicity.

^d^adjusted for age, and residents’ ethnicity.

^e^adjusted for age, year of death, citizenship, and CoD.

^f^adjusted for age, citizenship, and residents’ ethnicity.

## Discussion

Most CVD decedents during the studied period died out-of-hospital, with demographic and clinical factors significantly influencing the place of death. Individuals aged 18–45 years were more likely to die out-of-hospital compared to other age groups. Male decedents were slightly more likely to die out-of-hospital, albeit this association was not statistically significant. Being non-Saudi, including residents and pilgrims, were significant predictors of out-of-hospital deaths compared to Saudi citizens, and those who died from SCD had significantly higher odds of out-of-hospital deaths compared to those with ischemic heart diseases. The odds of out-of-hospital deaths decreased over the years, with a notable general decline from 2018 to 2023.

Although younger individuals had the highest proportion of out-of-hospital deaths 92.8% among those aged 18–45, our findings also revealed that the absolute number of deaths was substantially higher in older age groups, particularly those aged 61–75 years (3,552 deaths) and 76+ (2,625 deaths). This highlights that while the relative odds of dying outside the hospital are lower among older individuals, the population-level burden is greater in these groups. One explanation for the lower odds in older adults could be the presence of age-related complications and non-communicable diseases (NCDs), which often require hospital-based care. Chronic patients (e.g., with CVD) aged 61–75 years may have had more complex health conditions and a greater need for intensive care [30], and hospitalization helps cover comorbidities [31], consistent with studies indicating that older individuals with chronic illnesses are more frequently hospitalized at the end of life [32–34]. In contrast, out-of-hospital deaths in younger adults with CVD may reflect sudden and unexpected cardiac events [35], poor management (linked to undiagnosed hypertension or lack of preventive therapies) [36], reduced risk perception, and late help-seeking [37]. That the percentage differences in out-of-hospital deaths between age groups were relatively small yet produced larger variations in odds ratios likely reflects the model’s sensitivity to small changes in proportions, especially in large datasets, rather than indicating substantial clinical differences between age groups.

Increased out-of-hospital deaths among non-Saudi residents may plausibly be influenced by unmeasured social and health system barriers, such as differential access to healthcare, language challenges, and unfamiliarity with the local health system. For example, although non-Saudi residents working in the public sector have free healthcare access [38], and private sector expatriate workers have mandated employer-provided health insurance, which extends to non-working family members [39]. However, the extent and quality of this coverage can vary. These factors, along with out-of-pocket expenses, may discourage timely healthcare-seeking behavior. Language barriers (e.g., systemic gaps in multilingual support within healthcare settings) may also contribute to disparities in accessing emergency services, as attested by research across various contexts, including North America [40], Europe [41], and Asia [42]. Health literacy defined as the ability to obtain, process, and understand basic health information and services needed to make appropriate health decisions [43], may further impact healthcare access and decision-making. Although we did not directly assess these variables, previous studies suggest that inadequate health literacy is associated with higher cardiac mortality [44] and delayed in seeking care for acute myocardial infarction [45]. A study in Saudi Arabia estimated that 58.6% of the population (both Saudi and non-Saudi) has inadequate health literacy [46], highlighting the need for a more comprehensive understanding of multiple circumstantial social and health system barriers influencing access to healthcare services.

For pilgrims, the Saudi Ministry of Health provides free healthcare during the Hajj season. In addition, it establishes temporary medical facilities and deploys thousands of healthcare professionals to meet the increased demand. Despite these efforts, the massive influx of pilgrims can strain the healthcare system. An estimated 20–39.1% of Hajj pilgrims have chronic medical conditions [47,48], and CVD is the leading cause of mortality during Hajj [49]. In our dataset, deceased pilgrims had a mean age of 70 years (unpublished subgroup analysis), consistent with previous Hajj research [49]. Older adults are more vulnerable to CVD exacerbation [50], and the hot climate during summer Hajj seasons may further increase the risk of acute cardiac events [51]. These factors may contribute to the higher incidence of out-of-hospital deaths among pilgrims. However, further research is needed to more directly examine this association.

Out-of-hospital deaths remained high during 2020, which coincided with the COVID-19 pandemic. International reports have shown increased out-of-hospital cardiac arrests during this period, largely attributed to overwhelmed health systems and delayed emergency care [52–54]. In Saudi Arabia, however, strict lockdown measures were associated with reductions in overall NCDs mortality, while CVD continued to be the leading CoD [55,56]. The persistence of a high proportion out-of-hospital CVD deaths in 2020 may reflect reduced hospital access during lockdowns or patients’ reluctance to seek emergency care. The decline observed in later years may suggest the influence of broader healthcare reforms and the expansion of emergency services, though these should be considered contextual hypotheses rather than conclusions directly supported by our data.

A notably high proportion (93.6%) of deaths classified as SCD occurred in out-of-hospital settings in our dataset. This pattern raises concerns about potential misclassification, particularly given the lack of immediate medical assessment and diagnostic tools in these contexts. Although we did not directly assess data validity or misclassification, previous research in Saudi Arabia has shown that over 60% of death certificates may contain inaccurate or non-specific first-reported causes of death [57], suggesting that such challenges are common. In our context, the CoD for out-of-hospital deaths was typically based on physician-reported findings for individuals declared dead on arrival or documented through emergency medical services. In the absence of detailed medical history or post-mortem examination, physicians may rely on prior diagnoses or generalized classifications such as SCD. Moreover, cultural and logistical barriers to conducting autopsies [58], may further contribute to the frequent use of SCD as a default classification. Compared to other causes, such as ischemic heart disease, which are often better documented clinically, SCD appears disproportionately represented among out-of-hospital deaths, warranting further investigation.

Furthermore, our findings showed that individuals classified with SCD were significantly more likely to die outside of hospital compared to those with ischemic heart diseases. This pattern may reflect underlying issues such as delayed diagnosis and treatment due to low awareness of symptoms [59] or logistical barriers (e.g., transportation or taking time off work to visit healthcare centers). These challenges could lead to missed opportunities for timely care, increasing the risk of sudden and unexpected cardiovascular out-of-hospital deaths. Notably, SCD is more likely to occur out-of-hospital compared to ischemic heart diseases, entailing systemic diagnostic and ascertainment issues discussed above. The broad classification paradigm and absence of thorough clinical evaluations contribute to obscuring the true extent and nature of these conditions, complicating accurate CoD ascertainment.

The high rate of observed out-of-hospital deaths, particularly among high-risk populations (non-Saudis and SCD), highlights the need for continued strengthening of pre-hospital emergency care systems. Strategic efforts such as improving ambulances coverage, enhancing rapid-response cardiac capacity, and supporting dispatchers and first responders training may contribute to reducing pre-hospital mortality, especially in high-density areas where timely resuscitation can improve survival from sudden cardiac events.

Addressing disparities in out-of-hospital deaths between Saudi and non-Saudi residents is crucial. Expatriates working in lower-income sectors often face limited insurance coverage, which can delay access to medical care [60]. Policymakers should explore regulations that promote uniform and equitable insurance coverage across private and public sector workers, thereby reducing financial barriers to timely healthcare. Furthermore, enhancing multilingual medical services, culturally tailored health campaigns, and streamlined hospital admission protocols could improve expatriates’ engagement with healthcare facilities. Raising awareness about early recognition of cardiac symptoms among all residents is critical. Targeted educational campaigns that emphasize the importance of seeking prompt medical attention can help individuals at risk of silent or poorly managed CVD. Additionally, expanding community-based screening programs in workplaces and public spaces could facilitate early detection and timely intervention for high-risk individuals.

This study’s limitations include sourcing data from hospital-based medical registers with a limited number of variables. This constrained our ability to explore potentially significant factors such as socioeconomic status, geographic location, health literacy, access to healthcare services, and emergency response times. These unmeasured factors could have provided a more comprehensive understanding of the determinants of out-of-hospital deaths.

Second, the classification of causes of death, especially for out-of-hospital SCD, may be prone to misclassification, which could affect the accuracy of our findings as these represent a high proportion of deaths.

Third, in the hospital registers, pilgrims are categorized separately from Saudi citizens and non-Saudi residents. As a result, information about Saudis or non-Saudi residents who were also pilgrims is not captured, which may limit the precision of group comparisons and should be interpreted with caution.

Fourth, because the year 2023 included only two months of data, we conducted a sensitivity analysis excluding this year (S1 Table). The results were consistent with the main analysis, except that the CoD variable SCD was omitted due to collinearity, indicating that our findings are robust to this limitation.

Finally, although this study was conducted in Makkah, a setting characterized by its unique demographic diversity due to the religious Hajj and Umrah practices, this uniqueness may limit the generalizability of findings to other regions. However, it highlights the importance of conducting context-specific studies to understand better the determinants of the place of death in populations with such diverse and dynamic characteristics.

## Conclusion

Out-of-hospital deaths accounted for a substantial proportion of CVD-related mortality in Makkah City, influenced by key determinants such as age, citizenship status, and year and CoD. These findings underscore the need for tailored public health interventions that consider demographic and clinical disparities. Future research should explore the potential roles of healthcare access, medical insurance coverage, and systemic factors in shaping these outcomes, particularly among non-Saudi populations. Strengthening physician training in death certification and implementing standardized protocols for ascertaining causes of death may help reduce misclassification. The observed decline in out-of-hospital deaths over the study period may be linked to broader healthcare system reforms, although this requires further investigation.

## Supporting information

S1 Table Sensitivity analysis excluding 2023 data. (DOCX)

## Abbreviations

aOR: adjusted odds ratio
CoD: cause of death
CVD: cardiovascular disease
ICD-10: international classification of diseases 10
NCDs: non-communicable diseases
SCD: sudden cardiac death
