# Supplementary material for: Determinants of place of death among individuals with cardiovascular disease: A Saudi sub-national register study
Source: PLoS One. 2025 Oct 29;20(10):e0335669. doi: 10.1371/journal.pone.0335669 (PMC12571252; doi:10.1371/journal.pone.0335669)
Supplement: S1 Table — (DOCX) [file pone.0335669.s001.docx]

**S1 Table: Sensitivity analysis excluding 2023: adjusted odds ratios (95% CI) for associations between decedent characteristics and place of death, Makkah City, Saudi Arabia, 2018–2022**

| Characteristics | Main analysis  (2018–2023) | Sensitivity analysis (2018–2023) |
| --- | --- | --- |
| Age group (years) |  |  |
| 18-45 | *Ref* | *Ref* |
| 46-60 | 0.58 (0.43 – 0.78) | 0.55 (0.41 – 0.75) |
| 61-75 | 0.65 (0.49 – 0.86) | 0.64 (0.47 – 0.86) |
| 76+ | 0.76 (0.57 – 1.02) | 0.75 (0.56 – 1.02) |
| Sex |  |  |
| Female | *Ref* | *Ref* |
| Male | 1.14 (0.99 – 1.33) | 1.12 (0.99 – 1.31) |
| Year of death |  |  |
| 2018 | *Ref* | *Ref* |
| 2019 | 0.69 (0.55 – 0.86) | 0.69 (0.55 – 0.86) |
| 2020 | 0.84 (0.65 – 1.08) | 0.83 (0.64 – 1.07) |
| 2021 | 0.70 (0.54 – 0.91) | 0.69 (0.54 – 0.90) |
| 2022 | 0.38 (0.30 – 0.46) | 0.37 (0.30 – 0.46) |
| 2023 | 0.26 (0.18 – 0.39) | excluded |
| Citizenship |  |  |
| Saudi citizen | *Ref* | *Ref* |
| Non-Saudi resident | 2.05 (1.47 – 2.85) | 2.09 (1.47 – 2.97) |
| Pilgrim | 1.91 (1.38 – 2.66) | 1.85 (1.30 – 2.66) |
| Ethnicity |  |  |
| Arab | *Ref* | *Ref* |
| Non-Arab | 1.29 (0.95 – 1.74) | 1.30 (0.94 – 1.81) |
| CoD |  |  |
| Ischaemic heart diseases | *Ref* | *Ref* |
| SCD | 46.74 (37.35 – 58.48) | omitted |

Ref = Reference category; OR = Odds Ratio; CI = Confidence Interval.
**Note:** Cause of death (CoD) estimates for sudden cardiac death (SCD) could not be obtained in the sensitivity analysis due to collinearity and perfect prediction when 2023 was excluded.
